# Polyneuropathy, organomegaly, endocrinopathy, monoclonal band, and skin (POEMS) changes syndrome presenting with a pseudosensory level: a case report

**DOI:** 10.1186/s13256-019-2309-z

**Published:** 2019-12-27

**Authors:** Shiran Paranavitane, Lallindra Gooneratne, Thashi Chang

**Affiliations:** 1 University Medical Unit, National Hospital of Sri Lanka, Colombo, Sri Lanka; 2 Department of Pathology, Faculty of Medicine, University of Colombo, Colombo, Sri Lanka; 3 Department of Clinical Medicine, Faculty of Medicine, University of Colombo, Colombo, Sri Lanka

**Keywords:** POEMS, Castleman, Polyneuropathy, Pseudosensory, Lymphoproliferative

## Abstract

**Introduction:**

Polyneuropathy is a key feature of polyneuropathy, organomegaly, endocrinopathy, monoclonal band, and skin changes syndrome, which is a paraneoplastic manifestation of an underlying lymphoproliferative neoplasm. We report the first case of polyneuropathy, organomegaly, endocrinopathy, monoclonal band, and skin changes syndrome presenting with a pseudosensory level.

**Case presentation:**

A 59-year-old Tamil woman with long-standing diabetes mellitus and hypertension developed painless, progressive inguinal lymphadenopathy. A contrast-enhanced computed tomography scan showed mild hepatomegaly and intra-abdominal lymphadenopathy. A histological examination of an enlarged inguinal lymph node showed features of a plasma cell-type Castleman disease. She was treated with rituximab. Six months later, she developed gradually ascending numbness and weakness of both lower limbs. On examination, she had flaccid paraparesis (power 3/5) with a sensory level to pinprick at thoracic level 9. Joint position sense was preserved. Her cranial nerves and upper limbs were neurologically normal. Nerve conduction studies confirmed peripheral neuropathy with conduction slowing and a magnetic resonance imaging of her spine did not show cord or root compression. Serum protein electrophoresis showed a monoclonal band. A bone marrow biopsy showed a hypercellular marrow with 30% plasma cells. A repeat contrast-enhanced computed tomography scan showed sclerotic bony lesions involving multiple vertebrae in addition to mild hepatomegaly and intra-abdominal lymphadenopathy. Polyneuropathy, organomegaly, endocrinopathy, monoclonal band, and skin changes syndrome was diagnosed and she was treated with intravenously administered pulse therapy of dexamethasone and cyclophosphamide. After three cycles of treatment, she regained normal muscle power and sensation.

**Conclusions:**

Polyneuropathy in polyneuropathy, organomegaly, endocrinopathy, monoclonal band, and skin changes syndrome can present as a pseudosensory level.

## Introduction

Castleman disease is a lymphoproliferative disorder driven by proinflammatory cytokines [1]. It commonly presents with enlarged lymph nodes at one or more different sites. Castleman disease occurs in 11–30% of patients with polyneuropathy, organomegaly, endocrinopathy, monoclonal band, and skin changes (POEMS) syndrome which is considered to be a paraneoplastic manifestation of a lymphoproliferative disorder [2, 3]. Peripheral neuropathy is a common presenting feature and an essential criterion in the diagnosis of POEMS syndrome [4]. However, in approximately 5% of cases, patients present with a plasma cell dyscrasia such as Castleman disease prior to the development of neuropathy and other features of POEMS syndrome [4].

We report the case of a patient with Castleman disease who progressed to POEMS syndrome at which time she presented with a pseudosensory level.

## Case presentation

A 59-year-old Tamil woman with a 10-year history of adequately controlled diabetes mellitus and hypertension presented with a gradually enlarging painless lump in the right inguinal region over a period of 8 months. It was not associated with constitutional symptoms such as fever, weight loss, or loss of appetite.

On examination, the lump was consistent with an enlarged, non-tender, firm inguinal lymph node that was not attached to the underlying structures. She did not have any other palpable lymph nodes or organomegaly. She was not pale or icteric.

A contrast-enhanced computed tomography (CT) scan of her chest, abdomen, and pelvis showed mild hepatomegaly and intra-abdominal lymphadenopathy in addition to inguinal lymphadenopathy. Her complete blood count, liver functions, renal functions, inflammatory markers, and coagulation screen were within normal limits. Bone marrow aspiration and trephine biopsy were normal. An excisional biopsy of the enlarged lymph node showed evidence of Castleman disease of the plasma cell type with CD3 and Bcl-2 stained reactive pattern of follicles. She was treated with intravenously administered rituximab 375 mg/m^2^ weekly for 4 weeks. There was reduction in the size of her inguinal lymph nodes.

Six months later, she developed gradually ascending numbness and weakness of her lower limbs. These symptoms progressed in a symmetrical manner without upper limb or sphincter involvement. An examination revealed flaccid paraparesis with a proximal power of grade 4/5 and distal power of grade 3/5 associated with diminished lower limb deep tendon reflexes. Plantar responses were bilaterally flexor. She had impaired pinprick sensation up to thoracic 9 (T9) level with intact joint position sense and normal anal sphincter tone. There were no obvious spinal deformities or tender areas along her spine. A neurological examination of her cranial nerves and upper limbs did not reveal any deficits.

A nerve conduction study of her lower limbs diagnosed a moderately severe sensorimotor polyneuropathy with conduction slowing. Magnetic resonance imaging (MRI) of the thoracic and lumbosacral spine with gadolinium enhancement did not show compression of nerve roots or the thecal sac.

A summary of our patient’s hematological and biochemical investigations are shown in Table 1.

**Table 1:** Summary of the hematological and biochemical investigations

Investigation	Value	Reference range
White cell count	10,570	4000–11,000 cells/microL
Neutrophils	7870	2000–7000 cells/microL
Lymphocytes	1460	1000–4000 cells/microL
Hemoglobin	12.4	12–16 g/dL
Platelets	379,000	150,000–450,000/microL
Blood film	No abnormal cells seen. Within normal limits	
Ionized calcium	1.29	1.12–1.32 mmol/L
Serum creatinine	56	70–110 micromol/L
Sodium	134	135–145 mmol/L
Potassium	4.7	3.5–4.5 mmol/L
AST	32	< 40 U/L
ALT	30	< 35 U/L
Albumin	35	35–45 g/L
Globulin	40	30–40 g/L
ALP	106	30–120 U/L
ESR	11	< 20 mm/first hour
CRP	28	< 6 mg/L
TSH	4.461	0.35–4.78 mIU/L
Free T4	0.93	0.89–1.76 ng/dL
Rheumatoid factor	< 8	< 8 IU/L
Anti-nuclear antibodies	Negative	
HIV 1 and 2 antigen/antibody	Negative	
Blood culture	No growth	
Urine full report		
Proteins	1+	
Pus cells	2–4/HPF	
Red cells	1–2/HPF	
Casts	Nil	
Urine culture	No growth	

*ALP* alkaline phosphatase, *ALT* alanine transaminase, *AST* aspartate transaminase, *CRP* C-reactive protein, *ESR* erythrocyte sedimentation rate, *HIV* human immunodeficiency virus, *HPF* high-power field, *TSH* thyroid-stimulating hormone, *T4* thyroxine

Serum protein electrophoresis showed a faint monoclonal band in the fast gamma region without immunoparesis. However, urine protein electrophoresis was within normal limits. Immunofixation of the monoclonal band was not performed at the time due to unavailability. Bone marrow aspiration and trephine biopsy showed a hypercellular marrow with 30% plasma cells. A rectal biopsy showed normal rectal mucosa with focal ulceration. Congo red stain on the rectal biopsy did not reveal any amyloid deposits. A repeat contrast-enhanced CT scan of her chest, abdomen, and pelvis showed mild hepatomegaly, pericardial effusion, generalized subcutaneous tissue edema, multiple intra-abdominal lymphadenopathy, and multiple sclerotic bony lesions involving the thoracic and lumbar vertebral bodies, sternum, anterior ribs, and sacrum. A repeat MRI of her thoracolumbar spine was performed with gadolinium enhancement which showed altered signal intensity in multiple cervical and lumbar vertebral bodies in both T1 and T2 MRI sequences without destruction or collapse.

Based on the above findings, a diagnosis of POEMS syndrome was established. She was treated with six cycles of cyclophosphamide and dexamethasone, in addition to lower limb physiotherapy. Each 21-day cycle consisted of intravenously administered cyclophosphamide 750 mg/m^2^ infusion on day 1 and intravenously administered dexamethasone 40 mg daily on days 1 to 4.

Following three cycles of treatment, she demonstrated a remarkable improvement in her neurological deficits with recovery of muscle power and sensation to near normal.

## Discussion

We report the case of a patient with POEMS syndrome presenting with a sensory level. Loss of all modalities of sensation below one level on the trunk is pathognomonic of a lesion in the spinal cord. Rarely, lower motor neuron lesions affecting spinal nerves can present with a similar sensory loss [5]. A sensory level associated with a lower motor neuron lesion is known as a pseudosensory level. POEMS syndrome is characterized by polyneuropathy. Thus, the sensory level in our patient with POEMS syndrome was a pseudosensory level. POEMS syndrome has not been previously reported to present with a pseudosensory level.

The diagnosis of Castleman disease is made by histopathological examination of enlarged lymph nodes. It is a lymphoproliferative disorder which is mediated by proinflammatory cytokines such as interleukin-6 (IL-6) [1].

Our patient had multiple enlarged intra-abdominal and inguinal lymph nodes, which is in keeping with the diagnosis of multicentric Castleman disease [1]. Castleman disease is known to occur in isolation or progress to POEMS syndrome [4, 6]. Furthermore, it can also mimic lymphoproliferative neoplasms such as lymphoma and inflammatory disorders such as systemic lupus erythematosus [6, 7]. However, our patient’s bone marrow aspiration and trephine biopsy did not show evidence of lymphoma and her anti-nuclear antibodies were negative.

Multiple treatment modalities have been used in multicentric Castleman disease. These include rituximab, anti-IL-6 therapies such as tocilizumab, antivirals such as ganciclovir and zidovudine, and proteasome inhibitors such as bortezomib [8]. After confirming the diagnosis of multicentric Castleman disease of plasma cell type, she was treated with rituximab, to which there was a minimal response with reduction in the size of the inguinal lymph nodes.

Six months after the completion of rituximab therapy, this patient presented with lower motor neuron-type paraparesis and a pseudosensory level. Several possibilities were considered for this presentation; these included paraneoplastic peripheral neuropathy, chronic inflammatory demyelinating polyneuropathy (CIDP), rituximab-related peripheral neuropathy with an element of diabetic neuropathy, and POEMS syndrome.

The severity and rapidity of the peripheral neuropathy in a background of Castleman disease favored a paraneoplastic peripheral neuropathy. However, her bone marrow aspiration and trephine biopsy did not show evidence of tumor infiltration and contrast-enhanced CT of her chest, abdomen, and pelvis did not show solid organ tumors. The clinical progression of the neuropathy in this patient was not in keeping with classic CIDP and her nerve conduction studies ruled out this diagnosis. This patient’s clinical progression and nerve conduction findings were not in keeping with diabetes-related distal symmetric polyneuropathy alone. Hence, the possibility of an additional factor such as a drug-related neuropathy was considered. Rituximab is not well known to cause neuropathy. However, there have been reports of worsening of neuropathy after treatment with rituximab [9]. This has, however, been associated with anti- myelin-associated glycoprotein (MAG)-associated neuropathy and the worsening was noted to be transient and reversible after several weeks to months. Therefore, a severe neuropathy due to rituximab was unlikely.

Our patient developed polyneuropathy and was found to have a monoclonal band on serum protein electrophoresis. Further imaging showed mild hepatomegaly, pericardial effusion, generalized subcutaneous tissue edema, and multiple sclerotic bony lesions involving the thoracic and lumbar vertebral bodies, sternum, anterior ribs, and sacrum. These findings fulfilled the diagnosis of POEMS syndrome [3].

Her bone marrow aspiration and trephine biopsy showed 30% plasma cells. However, clonality of the plasma cells could not be established due to the inaccessibility to such a facility. POEMS syndrome is at present considered to be a paraneoplastic manifestation of an underlying plasma cell disorder [3]. Several factors differentiate POEMS syndrome from multiple myeloma such as prominent neuropathy, absence of renal involvement, sclerotic rather than lytic bone lesions, and elevated vascular endothelial growth factor (VEGF) levels. POEMS syndrome has shown better survival than multiple myeloma.

Although cytokines play an important role in the pathogenesis of POEMS syndrome, the current treatment modalities target the underlying clonal plasma cell disorder. Corticosteroids, thalidomide, lenalidomide, cyclophosphamide, bortezomib, bevacizumab, radiation, and allogenic stem cell transplantation have all been tried alone or in combination, with varying degrees of success [3].

Intravenously administered dexamethasone and cyclophosphamide pulse therapy was used in our patient. This regimen has demonstrated significant clinical improvement in at least 50% of patients [3]. Following three cycles of dexamethasone and cyclophosphamide pulses together with intense lower limb physiotherapy, there was improvement in the weakness and numbness.

## Conclusions

Castleman disease can, rarely, progress to POEMS syndrome despite treatment and therefore long-term follow-up is warranted. Furthermore, lower motor neuron syndromes, such as POEMS syndrome, can, rarely, present with a pseudosensory level and should not be confused with a myelopathy.

## Data Availability

All necessary data and material are provided.
